# *Wolbachia* and dengue virus infection in the mosquito *Aedes fluviatilis* (Diptera: Culicidae)

**DOI:** 10.1371/journal.pone.0181678

**Published:** 2017-07-21

**Authors:** Jéssica Barreto Lopes Silva, Debora Magalhães Alves, Vanessa Bottino-Rojas, Thiago Nunes Pereira, Marcos Henrique Ferreira Sorgine, Eric Pearce Caragata, Luciano Andrade Moreira

**Affiliations:** 1 Grupo Mosquitos Vetores: Endossimbiontes e Interação Patógeno-Vetor, Centro de Pesquisas René Rachou—Fiocruz, Belo Horizonte, Minas Gerais, Brazil; 2 Instituto de Bioquímica Médica, Universidade Federal do Rio de Janeiro, Rio de Janeiro, Brazil; International Atomic Energy Agency, AUSTRIA

## Abstract

Dengue represents a serious threat to human health, with billions of people living at risk of the disease. *Wolbachia pipientis* is a bacterial endosymbiont common to many insect species. *Wolbachia* transinfections in mosquito disease vectors have great value for disease control given the bacterium’s ability to spread into wild mosquito populations, and to interfere with infections of pathogens, such as dengue virus. *Aedes fluviatilis* is a mosquito with a widespread distribution in Latin America, but its status as a dengue vector has not been clarified. *Ae*. *fluviatilis* is also naturally infected by the *w*Flu *Wolbachia* strain, which has been demonstrated to enhance infection with the avian malarial parasite *Plasmodium gallinaceum*. We performed experimental infections of *Ae*. *fluviatilis* with DENV-2 and DENV-3 isolates from Brazil via injection or oral feeding to provide insight into its competence for the virus. We also examined the effect of the native *Wolbachia* infection on the virus using a mosquito line where the *w*Flu infection had been cleared by antibiotic treatment. Through RT-qPCR, we observed that *Ae*. *fluviatilis* could become infected with both viruses via either method of infection, although at a lower rate than *Aedes aegypti*, the primary dengue vector. We then detected DENV-2 and DENV-3 in the saliva of injected mosquitoes, and observed that injection of DENV-3-infected saliva produced subsequent infections in naïve *Ae*. *aegypti*. However, across our data we observed no difference in prevalence of infection and viral load between *Wolbachia*-infected and -uninfected mosquitoes, suggesting that there is no effect of *w*Flu on dengue virus. Our results highlight that *Ae*. *fluviatilis* could potentially serve as a dengue vector under the right circumstances, although further testing is required to determine if this occurs in the field.

## Introduction

Dengue virus (DENV) represents a serious threat to human health across the tropical regions of the world. There are four genetically distinct DENV serotypes, and infection can cause dengue, which has fever-like symptoms, or the comparatively rare severe dengue, which can lead to haemorrhaging and death. Subsequent infection with a different serotype greatly increases the risk that severe dengue will develop (WHO). There are an estimated 96 million cases that manifest clinically, each year [1], and nearly 4 billion people living at risk of infection [2], with severe, endemic transmission occurring in Latin America, and South and Southeast Asia.

In the last 40 years there has been an expansion in the incidence and prevalence of dengue cases [3]. This has occurred in conjunction with an increase in the geographic distribution of the mosquito vectors of the disease; the primary vector, *Aedes aegypti*, and the secondary vector *Aedes albopictus* [4]. Changes in climate, increased travel to disease-endemic areas, and increased deforestation and urbanization have served to bring humans and mosquitoes into contact more frequently, increasing the chance of viral transmission [1, 5–8]. Over the same time period there has been an increase in the prevalence of genetic resistance to commonly used insecticidal chemicals in mosquito populations, which has led to a decline in the efficacy of historically successful mosquito control programs [9, 10]. Urbanization and deforestation could also lead to a change in the dynamics of a pathogen transmission cycle, by increasing the frequency of contact between humans and other mosquito species that might become infected with the virus in the field [6, 11, 12].

*Aedes (Ochlerotatus) fluviatilis* (Lutz, 1904) is an anthropophilic species of mosquito native to Latin America, which lives in a wide variety of habitats, including urban and undeveloped areas [13]. *Ae*. *fluviatilis* has been described as being competent for infection with yellow fever virus, and was subsequently hypothesised as a potential vector for the disease in Latin America [14]. It has also been described as a good laboratory model for the avian malaria parasite *Plasmodium gallinaceum* [15]. However, little is known about the species’ ability to maintain infections with or transmit DENV.

*Wolbachia pipientis* is a maternally transmitted, bacterial endosymbiont that commonly infects many insect species [16]. *Wolbachia* strains are genetically diverse [17, 18], and maintain intricate relationships with their different host species [19, 20]. Many of these relationships are characterised by symbiont-induced manipulation of host reproduction, which allow the bacterium to persist in insect populations even in the face of fitness costs, and can permit the bacterium to spread through uninfected populations [21–24].

Many mosquito species are naturally infected by *Wolbachia*, including *Ae*. *fluviatilis*, which is infected by the *w*Flu strain [25, 26]. However, several key vector species, including most *Ae*. *aegypti* populations [27], are naturally uninfected. In these cases, stable infections have been generated by transinfection—the transfer of *Wolbachia* between species to form a stable infection [28]. *Ae*. *aegypti* has been independently transinfected with multiple *Wolbachia* strains [29–31], including the *w*Mel strain, originally found in the fruit fly *Drosophila melanogaster* [32]. *Wolbachia* transinfections in mosquitoes are strongly associated with pathogen interference—the ability to interfere with the replication and infection of pathogens such as the dengue and Zika viruses, and the human malarial parasite *Plasmodium falciparum* in mosquito tissues and saliva [32–35].

Transinfections in mosquitoes typically have a more severe physiological effect on their host than native infections, likely due to relatively novelty of infection, and subsequent activation of the host immune response [36]. Native infections are capable of altering host biology at the physiological and transcriptional level [25, 37], although *w*Flu has little apparent fitness cost [25]. They can also influence interactions between the host and pathogens, although not in the same way as transinfections. In *Ae*. *fluviatilis*, *w*Flu has been demonstrated to enhance infection with *P*. *gallinaceum*, increasing the intensity of infection at the oocyst stage [25]. A similar effect occurs for *Culex pipiens* and its native *Wolbachia*, *w*Pip, during infection with *Plasmodium relictum* [38]. This enhancement effect has also been observed in some instances of transient *Wolbachia* infection in mosquitoes [39–41], where *Wolbachia* is injected into adult mosquitoes to produce a somatic infection, but not in all cases [42, 43]. Enhancement has only been observed rarely, and never with a stable *Wolbachia* transinfection for a pathogen of humans. Unless this changes, the potential impact on mosquito control programs that involve *Wolbachia* is likely to be limited [44, 45]. However, given the enhancement of *P*. *gallinaceum* in *w*Flu-infected *Ae*. *fluviatilis*, we sought to determine if a similar effect occurred for DENV.

We performed a series of experimental infections with 2 different DENV isolates in order to examine the ability of *Ae*. *fluviatilis* to harbour infection with the virus. These experiments were performed on mosquitoes with and without the native *w*Flu infection in order to determine if the native *Wolbachia* infection of *Ae*. *fluviatilis* influenced infection with the virus. We also examined the effect of *w*Flu on the production of reactive oxygen species (ROS), as this had been linked to pathogen interference in other *Wolbachia*-infected insects [46, 47].

## Methods

### Mosquito rearing

Two lines of *Aedes fluviatilis* mosquitoes were involved in these experiments—one naturally infected with the *w*Flu *Wolbachia* strain (Flu), and the other where this infection had been cleared by treatment with tetracycline (Flu.Tet), as previously described [25]. Experiments were also performed on three lines of *Aedes aegypti—*one infected with the *w*Mel *Wolbachia* strain (Mel), and a second where the *w*Mel infection had been removed by treatment with tetracycline (Mel.Tet), as previously described [48]. These lines were previously backcrossed to a Brazilian genetic background [48], and were regularly outcrossed with *Wolbachia*-uninfected mosquitoes (WT) collected from Rio de Janeiro, RJ, Brazil, thereafter, in order to maintain genetic diversity [49]. All experiments took place at least two years after tetracycline treatments had finished. Saliva injection experiments were performed using the WT line. Microbiota re-colonization was performed as previously described [25, 49].

Flu and Flu.Tet larvae were reared at a density of approximately 400 larvae in 3L of distilled water and were fed 15–20 balls of Alcon Goldfish Colour fish food per day. Mel and Mel.Tet larvae were reared at a density of 150 larvae in 3L of distilled water, and were fed a ½ tetramin tropical tablet (Tetramin) per day. Pupae were moved to large cages in groups of 500. For experimental infections, adult females were transferred to small, cylindrical cages (diameter– 16cm, height– 18cm) containing approximately 50–70 individuals. Adults were maintained on 10% sucrose, which was changed daily for virus-infected mosquitoes, and three times per week for other cages. All mosquitoes were reared in a climate-controlled insectary (temperature—27 ± 1°C, RH - 70 ± 10%, photoperiod—12 hours light: dark).

### Dengue viruses & infection processes

Infections were performed using one of two DENV isolates. The DENV-2 isolate was isolated in Ribeirão Preto, SP, Brazil in 2000. The DENV-3 isolate, DENV-3 MG20 (375), was isolated in Contagem, MG, Brazil in 2013. Viruses were serially passaged in *Aedes albopictus* C6/36 cells, and infected supernatant harvested, titered via plaque forming assay, and then frozen at -80°C. Viral titres were 2.0x10^4^ pfu/mL for the DENV-2 isolate, and 1.9x10^6^ pfu/mL for the DENV-3. Virus aliquots were thawed only at the time of infection.

For injection experiments, viral stocks were injected, undiluted using a Nanoject II handheld injector (Nanoject) and glass capillaries. Each mosquito was injected intrathoracically with 69nL of virus (Approximate viral titre injected: DENV-2–1.4x10^0^, DENV-3–1.3x10^2^). Mosquitoes were collected at 5 days post-injection and stored at -80°C.

For oral infection experiments, viral stocks were mixed 1:1 with freshly drawn human blood, and fed to mosquitoes via glass feeders on a waterbath system. For the dilution experiment, one tube of frozen DENV-3 was thawed and diluted in L15 media, supplemented with 10% sterile, inactivated foetal bovine serum, to produce additional viral stocks at concentrations of, 1.9x10^4^, 1.9x10^3^ and 1.9x10^2^ pfu/mL. These were mixed with human blood and fed, as above. Immediately post-feeding, mosquitoes were screened visually for the presence of blood in the abdomen, and non-fed mosquitoes were discarded. Mosquitoes were collected at 7 or 14 days post-infection and stored at -80°C.

### Saliva collection & injection

Saliva was collected from mosquitoes that were infected with either DENV-2 or DENV-3 via injection, at 5 dpi, as previously described [34, 35]. Briefly, legs and wings were removed from anesthetized mosquitoes on ice, and mosquitoes were allowed 30 mins to expectorate into pipet tips containing 5μL of sterile, inactivated, foetal bovine serum and 30% sucrose (1:1), prepared fresh on the day of collection. These saliva samples were stored at -80°C and either assayed directly for the presence of DENV, or injected into naïve, wildtype, female *Aedes aegypti* mosquitoes, collected near Rio de Janeiro in 2016, as previously described [34]. *Ae*. *aegypti* mosquitoes were selected for injections given the propensity of that species to harbour infection with DENV. Each saliva was injected into 12–15 mosquitoes (volume injected: 138–276nL), which were collected at 5 dpi, and stored at -80°C. The presence or absence of DENV was determined by RT-qPCR for 7–8 injected mosquitoes per saliva sample.

### DENV quantification

DENV levels in mosquitoes from the *Ae*. *fluviatilis* and *Ae*. *aegypti* oral infection experiments, the DENV-3 dilution experiment, and the DENV-2 and DENV-3 injection experiments were quantified using TaqMan-based RT-qPCR, using a previously described protocol, primers and TaqMan probe [49]. Briefly, RNA from whole mosquito samples was extracted using the TRIzol protocol (Invitrogen), first-strand cDNA synthesis was performed using the M-MLV RT protocol (Promega), and qPCR was performed using a DENV general primer set and using a Viia 7 Real-Time PCR System (ThermoFisher Scientific). Absolute DENV levels were obtained by comparison with a serially diluted (10^7^ to 10^3^ copies) fragment of the PCR product, previously described [49], and were normalized per 1μg of total RNA. A minimum threshold of infection of 100 copies was applied to data across all experiments, as the 10^2^ standard did not amplify reliably.

Total RNA from saliva-injected mosquitoes was extracted using the High Pure Viral Nucleic Acid Kit (Roche), according to manufacturer’s instructions. The presence or absence of DENV was then determined via a duplex assay for DENV and the host RpS17 gene using the TaqMan Fast Virus 1-Step Master Mix (ThermoFisher Scientific), and run on a Lightcycler 96 (Roche). The qPCR mastermix contained the following components per reaction: 4.25μL water, 2μL RT-qPCR Mix, 0.5μL DENV primers (10μM), 0.1μL DENV probe (10μM—Texas Red), 0.5μL RpS17 primers (10μM), 0.1μL RpS17 probe (10μM—Hex), 0.05μL 200 X Reverse transcriptase enzyme. The water, RT-qPCR Mix, and enzyme were provided in the Fast Virus kit. The run profile was as follows: (1) 50°C for 10 mins, (2) 95°C for 30 sec, (3) 35 cycles of 95°C for 5 sec followed by 60°C for 30 sec, (4) 37°C for 30 sec.

### H_2_O_2_ quantification

H_2_O_2_ quantification was performed for 6 day-old Flu and Flu.Tet adult mosquitoes using the Amplex Red Hydrogen Peroxide/Peroxidase Assay Kit (ThermoFisher Scientific cat A22188). Three types of samples were collected across two experimental replicates: (1) whole mosquito, (2) midgut, (3) fat body, with the later two obtained by dissection using a light microscope in ice cold PBS. Total samples collected ranged from 12–22 per treatment. Samples were collected on ice, and then immediately processed according to manufacturer’s instructions, as described elsewhere [50]. H_2_O_2_ levels were quantified using a microplate reader (SpectraMax M5, Molecular Devices, Sunnyvale, CA, USA), with an excitation wavelength of 530nm and an emission wavelength of 590nm, and values compared against a standard curve generated from H_2_O_2_ samples of known concentration. Mosquitoes in these assays were not infected by DENV.

### Data analysis

DENV prevalence of infection was compared using Fisher’s exact test. For saliva-injected mosquitoes, prevalence of infection was compared on the individual mosquito level. Comparisons of viral load were only made for samples that tested positive for DENV. These data were all non-parametrically distributed, and were thus compared using Mann-Whitney U tests. No viral load comparisons were made for treatments with fewer than 3 positive samples, in order to satisfy the requirements of the Mann-Whitney U test. For these pairwise comparisons, only statistics for prevalence are presented. H_2_O_2_ data were all normally distributed, and were compared using unpaired t tests. All analyses were performed using Graphpad Prism V 6.0g. Figures were prepared using Graphpad Prism and Microsoft PowerPoint for Mac 2011.

### Human ethics

Human blood was drawn from willing adult volunteers by trained medical personnel after obtaining written consent. This process was conducted according to established guidelines, and approved by The Committee for Ethics in Research (CEP)/ FIOCRUZ (License—CEP 732.621), and performed in accordance with the Brazilian laws (196/1996 and 01/1988), which govern human ethics issues in scientific research in compliance with the National Council of Ethics in Research (CONEP).

## Results

### DENV injections

**Fig 1:**
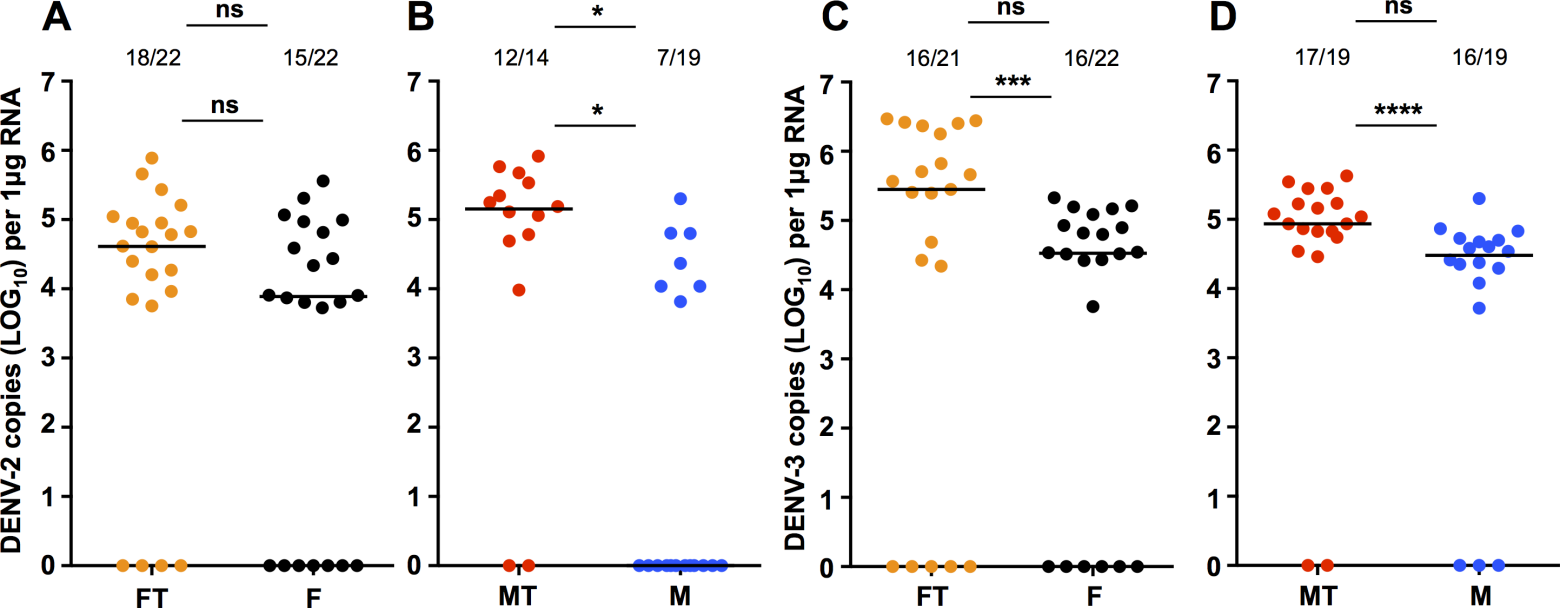
DENV-2 or DENV-3 infection via intrathoracic injection in *Aedes fluviatilis* and *Aedes aegypti*. Prevalence of infection and DENV load for *Ae*. *fluviatilis* and *Ae*. *aegypti* mosquitoes 5 days after intrathoracic injection with DENV-2 **(A&B)** or DENV-3 **(C&D)**, as determined via RT-qPCR with absolute quantification. FT (orange)—Flu.Tet. F (black)—Flu. MT (red)—Mel.Tet. M (blue)—Mel. Prevalence data analysed by Fisher’s exact test. DENV load data analysed by Mann-Whitney U test. ns = *P* > 0.05, * = *P* < 0.05, *** = *P* < 0.001, **** = *P* < 0.0001. Black lines represent treatment medians.

We injected adult female *Ae*. *fluviatilis* and *Ae*. *aegypti* with either DENV-2 or DENV-3, collected these mosquitoes 5 days later, and then quantified levels of DENV using RT-qPCR. For *Ae*. *fluviatilis* mosquitoes injected with DENV-2 (Fig 1A), we observed that 15/22 Flu mosquitoes, and 18/22 Flu.Tet mosquitoes were positive for the virus, and that there was no effect of *w*Flu infection on prevalence of infection (Fisher’s exact test; *P* = 0.4876). Likewise, we saw no difference in viral load between Flu and Flu.Tet mosquitoes (Mann-Whitney U test; *U* = 89, *P* = 0.5045). For *Ae*. *aegypti*, we observed a decrease in DENV-2 prevalence associated with *w*Mel infection (Fig 1B), as 8/20 Mel mosquitoes were positive for the virus compared with 12/14 Mel.Tet mosquitoes (Fisher’s exact test; *P* = 0.0128). Likewise, DENV-2 viral load was also decreased for Mel mosquitoes (Mann-Whitney U test; *U* = 16, *P* = 0.0283).

For *Ae*. *fluviatilis* mosquitoes challenged with DENV-3 by injection (Fig 1C), we saw 16/22 Flu and 17/22 Flu.Tet mosquitoes became infected (Fisher’s exact test; *P* = 1.000). However, in this case *w*Flu infection led to a significant decrease in DENV load (Mann-Whitney U test; *U* = 38, *P* = 0.0004). We observed no decrease in prevalence for *Ae*. *aegypti* injected with DENV-3 (Fig 1D), with 16/19 Mel mosquitoes and 17/19 Mel.Tet mosquitoes infected (Fisher’s exact test; *P* = 1.000). However, *w*Mel infection did significantly reduce DENV-3 load amongst infected mosquitoes (Mann-Whitney U test; *U* = 21.5, *P* < 0.0001).

### DENV oral infections

**Fig 2:**
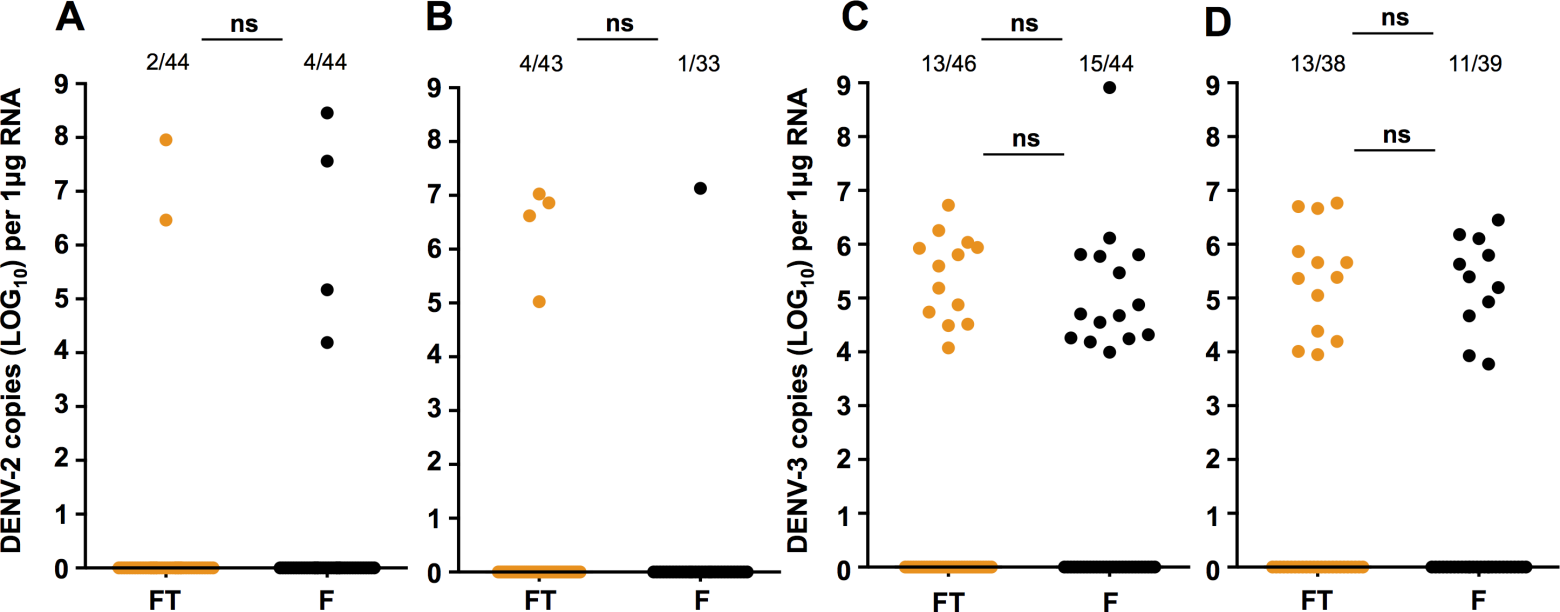
DENV-2 or DENV-3 oral infection in *Aedes fluviatilis*. Prevalence of infection and DENV load for *Ae*. *fluviatilis* orally challenged with DENV-2 at 7 dpi **(A)** and 14 dpi **(B)**, or DENV-3 at 7 dpi **(C)** and 14 dpi **(D)**, as determined via RT-qPCR with absolute quantification. Data represent 2 independent infections. FT (orange)—Flu.Tet. F (black)—Flu. Prevalence data analysed by Fisher’s exact test. DENV load data analysed by Mann-Whitney U test. ns = *P* > 0.05. Black lines represent treatment medians.

We then orally infected Flu and Flu.Tet mosquitoes with either DENV-2 or DENV-3. Infection with the DENV-2 isolate produced only a very small number of positives from either line. Accordingly, we examined data from both experimental infections together. At 7dpi (Fig 2A), we saw that 4/40 Flu and 2/42 Flu.Tet mosquitoes became infected, but this did not amount to a significant difference in prevalence (Fisher’s exact test; *P* = 0.6763). At 14dpi (Fig 2B), 1/32 Flu mosquitoes, and 4/39 Flu.Tet mosquitoes became infected, and again these proportions were not significantly different (Fisher’s exact test; *P* = 0.3806). Numbers of infected mosquitoes were not sufficient to compare viral load.

Oral infection with the DENV-3 isolate produced a slightly higher rate of infection. Across the two experiments, we saw prevalence of infection of 15/29 for Flu mosquitoes and 13/33 for Flu.Tet mosquitoes at 7dpi (Fig 2C; Fisher’s exact test; *P* = 0.6503), and 11/28 for Flu and 13/25 for Flu.Tet mosquitoes at 14dpi (Fig 2D; Fisher’s exact test; *P* = 0.6280). Critically, there was no difference in prevalence due to *Wolbachia*. Likewise, we saw no difference in viral load between the two mosquito lines at either time point (Mann-Whitney U test; 7dpi—*U* = 71, *P* = 0.2352: 14dpi—*U* = 66, *P* = 0.7648).

**Fig 3:**
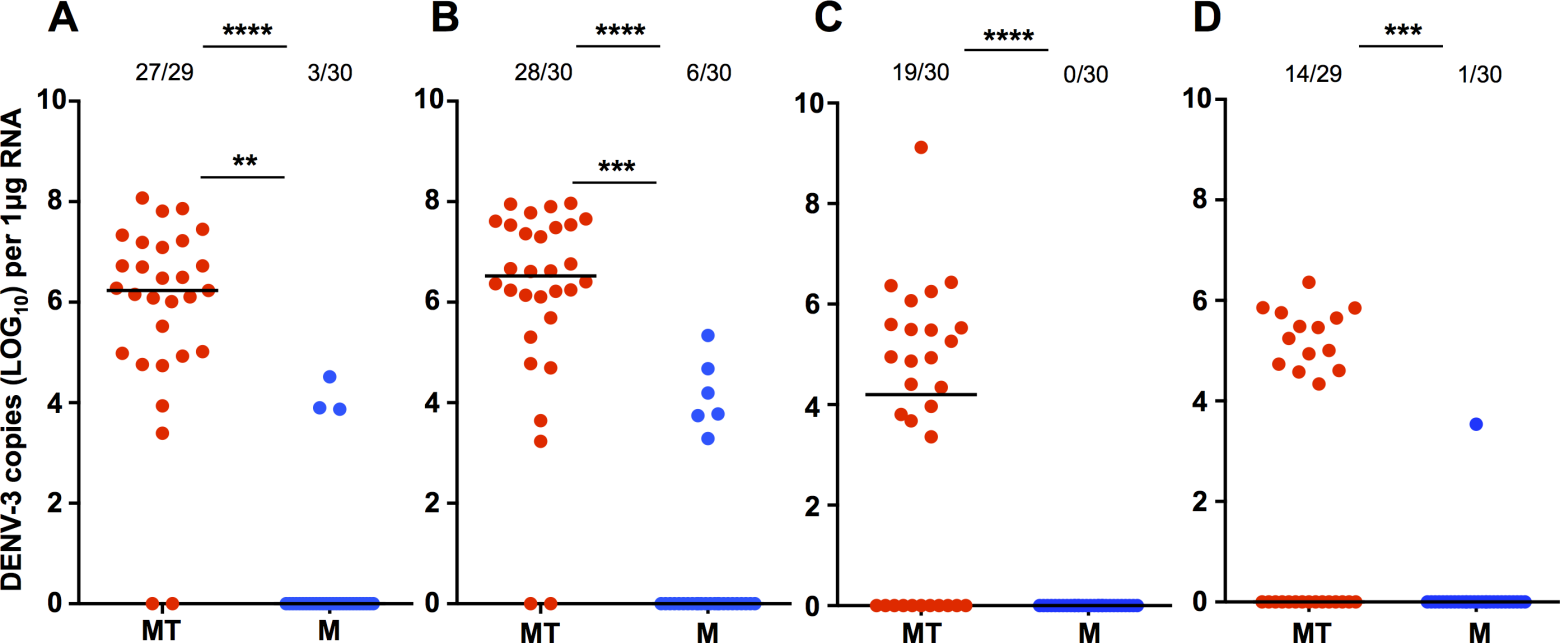
DENV-3 oral infection in *Aedes aegypti*. Prevalence of infection and DENV load for *Ae*. *aegypti* orally challenged with DENV-3 in experiment 1 (7 dpi **(A)** and 14 dpi **(B)**), and in experiment 2 (7 dpi **(C)** and 14 dpi **(D)**), as determined via RT-qPCR with absolute quantification. MT (red)—Mel.Tet. M (blue)—Mel. Prevalence data analysed by Fisher’s exact test. DENV load data analysed by Mann-Whitney U test. ** = *P* < 0.01, *** = *P* < 0.001, **** = *P* < 0.0001. Black lines represent treatment medians.

We also examined DENV infection in *Ae*. *aegypti*, as a point of comparison. As the DENV-2 isolate did not infect *Ae*. *fluviatilis* well, we only performed experiments with the DENV-3 isolate. At 7dpi in the first experiment (Fig 3A), 27/29 Mel.Tet became infected compared to 3/30 Mel mosquitoes (Fisher’s exact test; *P* < 0.0001). Those Mel mosquitoes that became infected had a significantly lower viral load than Mel.Tet mosquitoes (Mann-Whitney U test; *U* = 4, *P* = 0.0054). At 14dpi (Fig 3B), 28/30 Mel.Tet were infected compared to 6/30 Mel mosquitoes (Fisher’s exact test; *P* <0.0001), and viral load was again significantly lower in the infected Mel mosquitoes (Mann-Whitney U test; *U* = 14, *P* = 0.0006).

In the second *Ae*. *aegypti* oral infection experiment, 19/30 Mel.Tet mosquitoes and 0/30 Mel mosquitoes became infected at 7dpi (Fig 3C). Prevalence of infection was significantly lower amongst Mel mosquitoes than either Mel.Tet (Fisher’s exact test; *P* < 0.0001). At 14dpi (Fig 3D), 14/29 Mel.Tet mosquitoes and 1/30 Mel mosquitoes became infected. Prevalence of infection was significantly lower amongst Mel mosquitoes than Mel.Tet mosquitoes (Fisher’s exact test; *P* = 0.0002). As less than 3 Mel mosquitoes became infected at either time point, comparison of viral load was not performed.

In a further oral feeding assay (S1 Fig), we offered the DENV-3 isolate at dilutions of 1.9x10^6^ (conc. 1), 1.9x10^4^ (conc. 2), 1.9x10^3^, and 1.9x10^2^ pfu/mL. The prevalence of infection across the lower concentrations was less than 5% for all treatments, and the data were not considered for further analysis. For *Ae*. *fluviatilis*, 13/22 Flu mosquitoes became infected at conc. 1 at 7 dpi, compared to 3/14 Flu.Tet (Fisher’s exact test; *P* = 0.0407), however, we saw no effect on viral load (Mann-Whitney U test; *U* = 15, *P* = 0.6107). Six Flu and 2 Flu.Tet mosquitoes became infected at conc. 2 for the same time point, but this was not a significant difference in prevalence (Fisher’s exact test; *P* = 0.2127). At 14dpi, 9/18 Flu samples, and 7/17 Flu.Tet samples became infected after feeding on conc. 1 (Fisher’s exact test; *P* = 0.7380), with no significant difference in viral load (Mann-Whitney U test; *U* = 31, *P* > 0.999). While at conc. 2 1/18 Flu, and 2/18 Flu.Tet samples were positive for DENV-3 (Fisher’s exact test; *P* = 1.000). For *Ae*. *aegypti*, no Mel mosquitoes became infected at any time point, for any concentration. There was a significant decrease in prevalence associated with *w*Mel infection at conc. 1, at both 7 and 14dpi (Fisher’s exact test; *P* < 0.0001). For conc. 2, Mel.tet prevalence was 0/15 and 2/11 at 7 and 14dpi, respectively.

### Salivation assays

**Fig 4:**
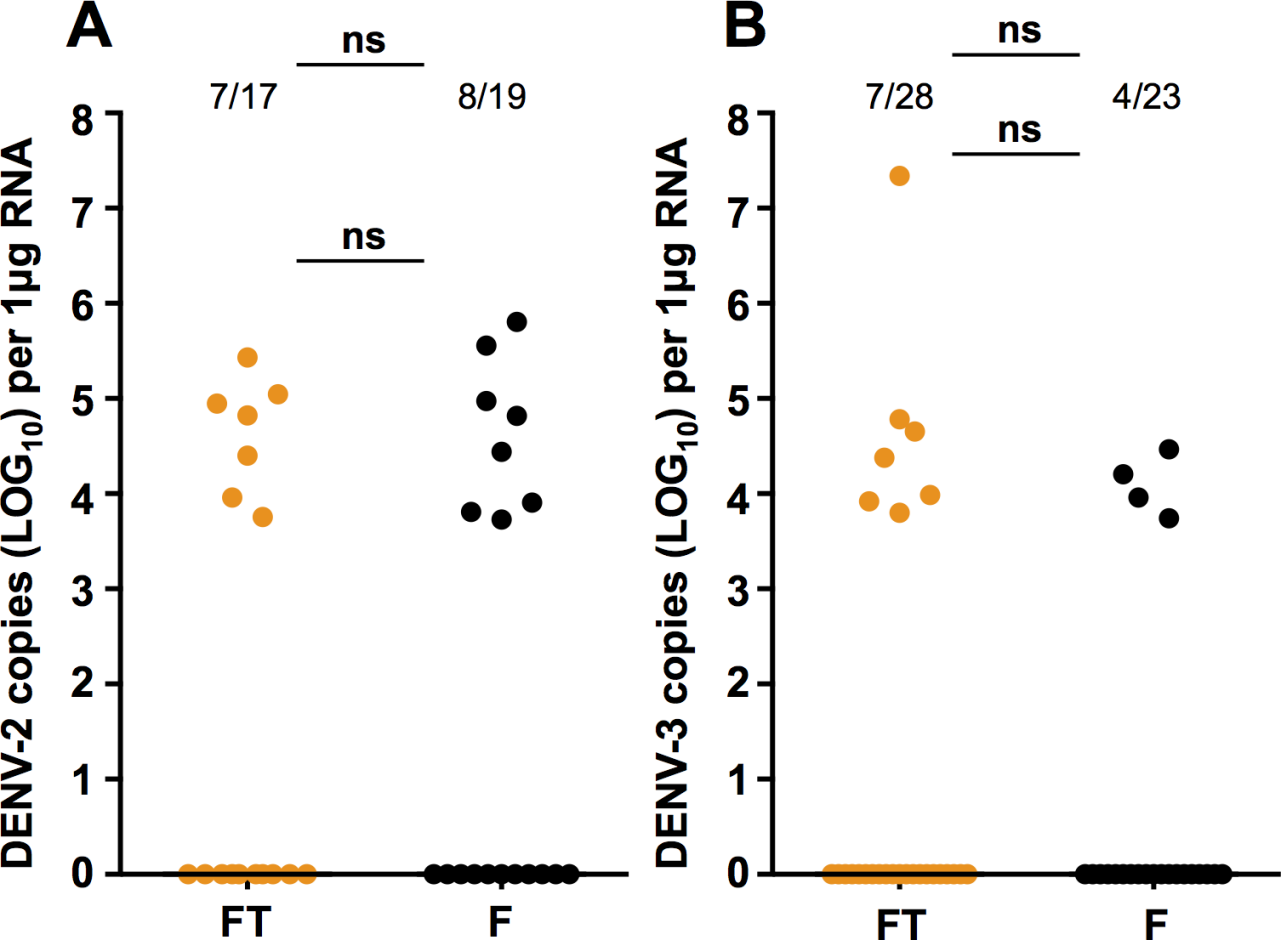
DENV-2 or DENV-3 load in the saliva of *Aedes fluviatilis*. Prevalence of infection and DENV load in saliva collected from *Ae*. *fluviatilis* mosquitoes injected with DENV-2 **(A)** or DENV-3 **(B)**, as determined via RT-qPCR with absolute quantification. FT (orange)—Flu.Tet. F (black)—Flu. Prevalence data analysed by Fisher’s exact test. DENV load data analysed by Mann-Whitney U test. ns = *P* > 0.05. Black lines represent treatment medians.

We quantified levels of DENV directly in saliva collected from Flu and Flu.Tet mosquitoes that had been injected with either the DENV-2 (Fig 4A) or DENV-3 isolates (Fig 4B). For DENV-2, 8/19 Flu saliva and 7/17 Flu.Tet saliva were positive for the virus (Fisher’s exact test; *P* = 1.000). While for DENV-3, 4/23 Flu saliva, and 7/28 Flu.Tet saliva were positive (Fisher’s exact test; *P* = 0.7338). Comparison of viral load in these saliva samples revealed that there was no significant of *w*Flu infection for either DENV-2 (Mann-Whitney U test; *U* = 27, *P* = 0.9551) or DENV-3 (Mann-Whitney U test; *U* = 9, *P* = 0.4121).

**Fig 5:**
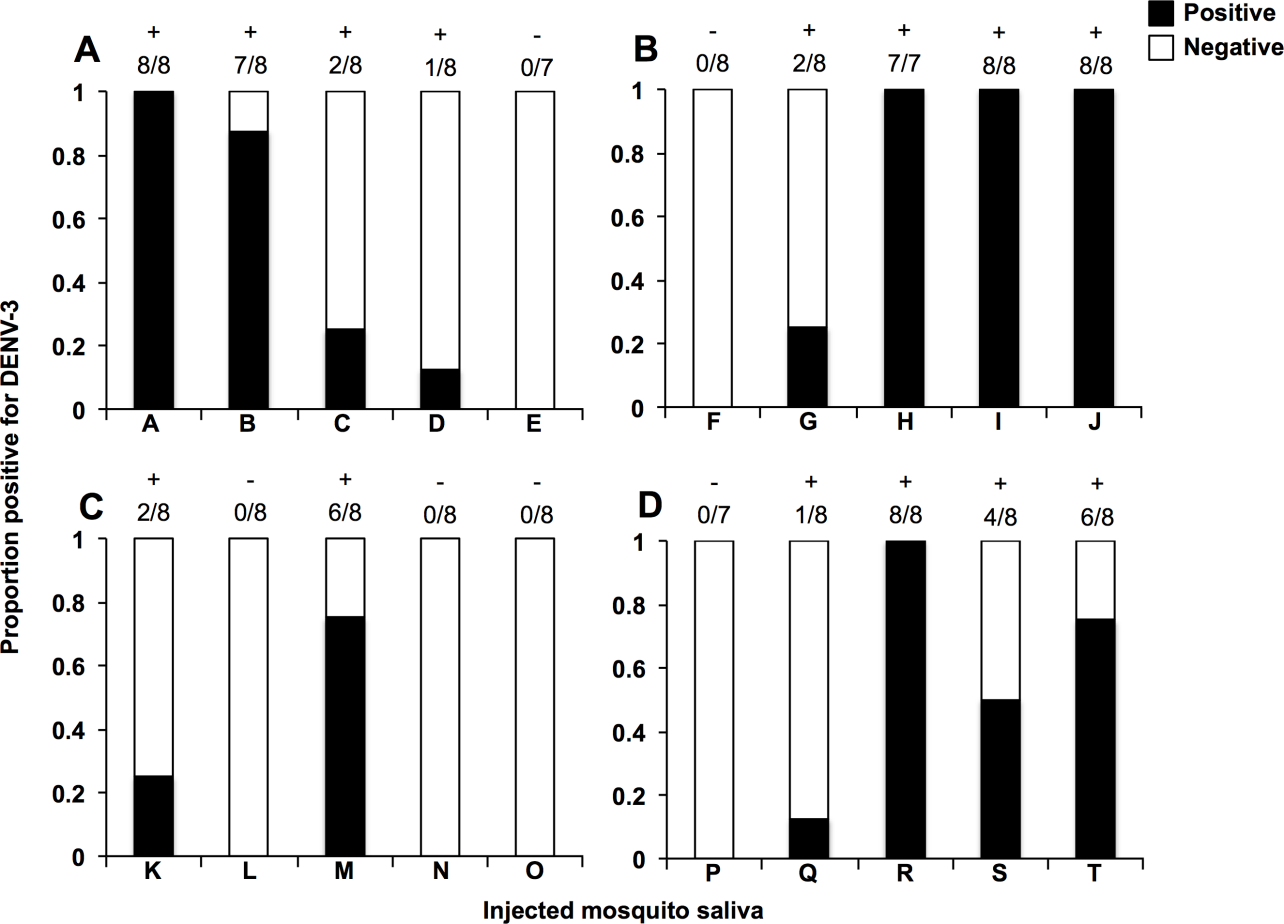
DENV-3 infectivity after the injection of saliva from infected *Aedes fluviatilis* and *Aedes aegypti* into naïve mosquitoes. The presence of absence of DENV was determined in 7–8 individual mosquitoes injected with saliva collected from DENV-3-infected Flu.Tet **(A)**, Flu **(B)**, Mel.Tet **(C)**, or Mel **(D)** mosquitoes, as determined by RT-qPCR. Each letter code (A-T) represents a single saliva sample. In each bar, black represents the proportion positive for DENV, and white represents the proportion where DENV was not detected. + = saliva that produced a subsequent infection.— = saliva that produced no subsequent infection.

We then took 5 saliva samples collected from Flu (Fig 5A), Flu.Tet (Fig 5B), Mel (Fig 5C), and Mel.Tet (Fig 5D) mosquitoes that had been injected with DENV-3, and injected these into WT mosquitoes. For saliva from *Ae*. *fluviatilis*, 4/5 Flu and 4/5 Flu.Tet saliva produced subsequent infections, at an average infection rate of 65% and 45%, respectively. We saw no significant difference in the overall prevalence of saliva-injected mosquitoes (Fisher’s exact test; *P* = 0.1716). For saliva from *Ae*. *aegypti*, 2/5 Mel and 4/5 Mel.Tet saliva produced subsequent infections, at average infection rates of 20% and 47.5%, respectively. In this instance there was a decrease in overall prevalence associated with *w*Mel infection in the mosquitoes that produced the saliva (Fisher’s exact test; *P* = 0.0093).

### Reactive oxygen species

**Fig 6:**
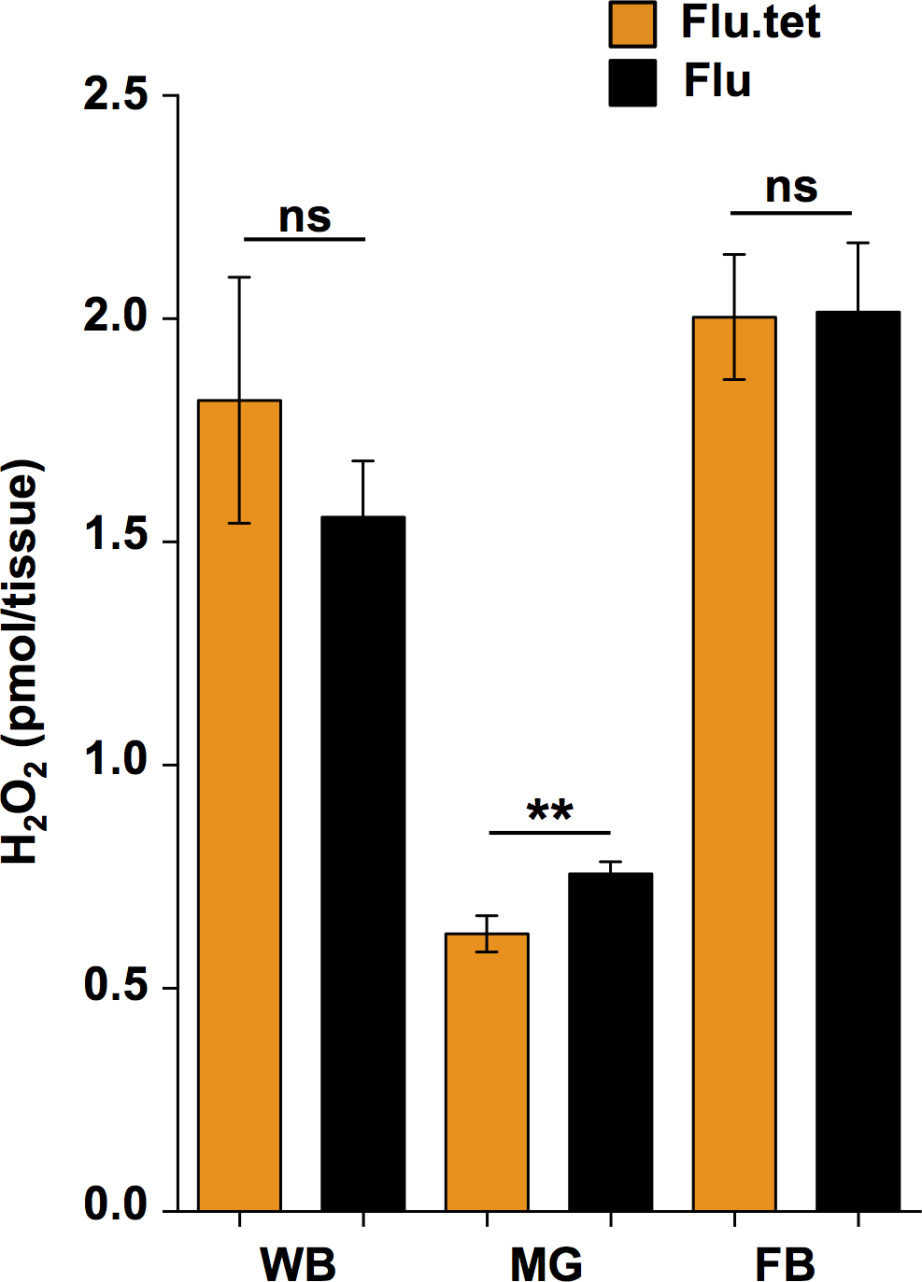
H_2_O_2_ levels in *Aedes fluviatilis*. Levels of H_2_O_2_ in the whole bodies (WB), midguts (MG) and fat bodies (FB) of Flu.Tet (orange) and Flu (black) mosquitoes, quantified using the Amplex Red Hydrogen Peroxide/Peroxidase Assay Kit with a fluorometric plate reader. Data were compared using unpaired t tests. ns = *P* > 0.05, ** = *P* < 0.01.

We compared levels of ROS (H_2_O_2_) in Flu and Flu.Tet mosquitoes for the whole mosquito, midgut or fat bodies (Fig 6). We saw that H_2_O_2_ levels were not significantly different in whole mosquitoes (Unpaired t test; *t* = 0.8635, *P* = 0.3972) or in the fat body (Unpaired t test; *t* = 0.0514, *P* = 0.9594), however, H_2_O_2_ levels were slightly, but significantly elevated in the midguts of Flu mosquitoes compared to Flu.Tet (Unpaired t test; *t* = 2.739, *P* = 0.0090). Mean (± s.e.m.) H_2_O_2_ levels were 0.7563 ± 0.027 for Flu mosquitoes, and 0.6222 ± 0.041 for Flu.Tet mosquitoes.

## Discussion

Our results from the experimental infection of *Ae*. *aegypti* with DENV-3 showed high prevalence of infection and DENV load in Mel.Tet mosquitoes, and a strong pathogen interference effect in Mel mosquitoes. This persisted even when the mode of infection was via injection. Several recent papers have demonstrated that *w*Mel interferes with Zika virus and chikungunya virus in *Ae*. *aegypti* populations from Brazil and Colombia [34, 51, 52]. Our results provide further evidence that the pathogen interference phenotype operates effectively in Latin American *Ae*. *aegypti*. These results also suggested that the DENV-3 isolate that we used was sufficiently infectious to serve as a good model strain for examining DENV infection in *Ae*. *fluviatilis*. It should be noted that the our results were obtained using whole mosquito samples, and thus likely underreport the blocking effects of *w*Mel, as this strain is a strong blocker of disseminated and salivary infection [34].

### Can *Aedes fluviatilis* transmit DENV?

We performed experimental infections of *Ae*. *fluviatilis* mosquitoes with DENV-2 and DENV-3 isolates via oral feeding and intrathoracic injection. With the oral feeding experiments we observed very low infection rates for the DENV-2 isolate (3–10%), with this likely being due to a low viral titre (2.0x10^4^ pfu/mL). For the DENV-3 isolate, we observed combined infection rates of 27–44% at 7dpi, and 35–37% at 14dpi for both Flu and Flu.Tet mosquitoes, across 3 experiments. These infection rates were far lower than what we observed when the Mel.Tet line (*Ae*. *aegypti* without a *Wolbachia* infection) was infected with the same virus, where overall infection rates were 78% at 7dpi and 71% at 14dpi, across 2 experiments. We observed higher rates of infection after *Ae*. *fluviatilis* mosquitoes were infected by intrathoracic injection, with an overall infection rate of 68–82% for DENV-2, and 73–76% for DENV-3. These values were more similar to the infection rates we observed for the Mel.Tet line (DENV-2: 86%, DENV-3: 89%). These data suggest that *Ae*. *fluviatilis* can become infected by DENV after experimental infection in the laboratory. As the titre of the DENV isolates used in our experiments was somewhat low, it is possible that *Ae*. *fluviatilis* may prove to be more susceptible to infection with different DENV isolates, or those fed at a higher titre.

We also examined the saliva of *Ae*. *fluviatilis* mosquitoes for the presence of DENV using two different techniques, after the mosquitoes had been infected with either DENV-2 or DENV-3 by injection. When viral load was quantified directly in the saliva, we observed that 41–42% of saliva tested positive for DENV-2, and 17–25% were positive for DENV-3, for Flu and Flu.Tet mosquitoes. When these saliva were injected into WT *Ae*. *aegypti* we were also able to detect DENV infection in a high percentage of mosquitoes. As virus load in these mosquitoes was quantified 5 days post-injection, this may indicate that the virus was able to replicate, suggesting that infectious vius can be found in the saliva of *Ae*. *fluviatilis*.

Our results provide some evidence that *Ae*. *fluviatilis* could potentially transmit DENV under the right circumstances. However, we state this with the acknowledgment of several caveats. Firstly, all of our data showing DENV infection in saliva were obtained after intrathoracic injection and not oral feeding. Given the lower prevalence of infection associated with oral feeding this may suggest that that the midgut is an important barrier to DENV infection in *Ae*. *fluviatilis*, and it is possible that this species does not possess some of the receptors for DENV that are present in other mosquito species [53]. Secondly, the *Ae*. *fluviatilis* lines we used are not necessarily representative of the species in the field, given that the colony has been maintained in the laboratory since 1975 [25]. It is also unclear how likely it is that *Ae*. *fluviatilis* in the field would encounter and bite a human infected with dengue, or if a DENV-infected *Ae*. *fluviatilis* would be capable of transmitting the virus to a new human host. To that end, we suggest that future research on this topic should involve a survey of wild *Ae*. *fluviatilis* populations for the presence of DENV, and that saliva be collected from further experimental infections where field populations of *Ae*. *fluviatilis* are orally infected with fresh, recently circulating DENV strains.

### Influence of *Wolbachia*

With respect to a potential effect of *w*Flu on DENV infection, we observed high infection rates in Flu mosquitoes across multiple experiments, for both modes of infection. However, we saw similar results for Flu.Tet mosquitoes. In one oral feeding experiment there was increased prevalence of infection associated with *w*Flu, and in one injection experiment, there was decreased viral load for DENV-3 associated with *w*Flu. For all other experiments we saw no effect of *w*Flu infection on prevalence or intensity of infection. At the saliva level, there was slightly higher prevalence of infection for mosquitoes injected with saliva from DENV-infected Flu mosquitoes than Flu.Tet mosquitoes, although this difference was not significant. Taken together these results provide no evidence of a consistent effect of *w*Flu on DENV, which stands in contrast to what was previously observed for *P*. *gallinaceum* [25].

The ability of a *Wolbachia* strain to interfere with pathogens has been strongly linked to high bacterial density, and high *Wolbachia* density in mosquitoes is typically associated with transinfections, rather than native associations [29, 32]. In *Ae*. *fluviatilis*, *w*Flu is most abundant in the ovaries, which facilitates maternal transmission, but it is much less abundant in somatic tissues than transinfections, including that of *w*Mel [25, 26, 32]. Given these observations it was not surprising that we observed no pathogen interference effect of *w*Flu.

### ROS induction

ROS induction (higher levels of H_2_O_2_ in the presence of *Wolbachia* infection) in mosquitoes is a trait that is most commonly associated with *Wolbachia* transinfections [19, 46]. We recently demonstrated that this induction effect occurs for *w*Mel-infected *Ae*. *aegypti* [49], while a similar effect has been observed for *w*AlbB-infected *Ae*. *aegypti* [46]. However, the effect has not been associated with native infections in mosquitoes. The reason for this is hypothesized to be due to a longer period of host-symbiont co-adaptation, and potentially restored redox homeostasis in native infections [54]. Our data suggest that *w*Flu does cause a moderate level of ROS induction in *Ae*. *fluviatilis*, but only in the midgut. Our recent transcriptomic profile of *Ae*. *fluviatilis* revealed *w*Flu-induced changes in the expression of several genes linked to redox process [37]. However, the fact that we observed no difference in whole mosquitoes, or in fat bodies, suggests that *w*Flu is unlikely to have a major impact on redox homeostasis, which could be indicative of extensive co-evolution between host and symbiont [54]. While the small scale ROS induction that we observed in the midguts was likely insufficient to have a significant effect on a pathogen.

ROS induction has also been shown to occur with some native *Wolbachia* infections in *Drosophila*, and its presence correlates well with the presence of pathogen interference [47]. But, higher ROS levels are not found in all *Wolbachia*-host associations where pathogen interference has been observed [55], and it is still unclear how *Wolbachia*-induced changes in ROS contribute to the phenotype. Not all mosquito midgut cells become infected with DENV, and there are foci of infection [56, 57]. It is unclear if the *Wolbachia*-induced increase in ROS is homogeneously or heterogeneously distributed through tissues and cells. If the latter were true, cells with higher ROS levels could become more resistant to the virus, leaving fewer “entry points” available.

## Conclusions

We have demonstrated that the mosquito *Ae*. *fluviatilis* can become infected with DENV after intrathoracic injection or oral feeding, although at lower rates than what is observed with *Ae*. *aegypti*, a proven vector of DENV in the field. We observed that DENV can be detected in the saliva of *Ae*. *fluviatilis* after viral challenge via injection, and that this virus is likely infectious, given that it can be used to infect naïve *Ae*. *aegypti*. The vector status of this species is still uncertain, but our results suggest that it could potentially play a role in DENV transmission. Our results also indicate that the native *Wolbachia* infection of *Ae*. *fluviatilis* does not influence DENV infection, but does increase ROS levels in the host midgut, with neither of these observations being particularly unexpected for a native *Wolbachia*-host association.

## Supporting information

S1 Fig DENV-3 titres in *Aedes aegypti* and *Aedes fluviatilis* fed either 10^6^ or 10^4^ pfu per mL. Prevalence of infection and DENV load for *Ae*. *fluviatilis* at 7 **(A)** and 14dpi **(B)**, and *Ae*. *aegypti* at 7 **(C)** and 14dpi **(D)**, as determined via RT-qPCR with absolute quantification. The figure shows data for two DENV-3 concentrations that were fed to mosquitoes: 1.9 x10^6^ pfu/mL (1), and 1.9 x 10^4^ pfu/mL (2). FT (orange)—Flu.Tet. F (black)—Flu. MT (red)—Mel.Tet. M (blue)—Mel. Prevalence data analysed by Fisher’s exact test. DENV load data analysed by Mann-Whitney U test. ns = *P* > 0.05, * = *P* < 0.05, **** = *P* < 0.0001. Black lines represent treatment medians.
(TIFF)
