# Supplementary figures and images for: Wolbachia and dengue virus infection in the mosquito Aedes fluviatilis (Diptera: Culicidae)
Source: PLoS One. 2017 Jul 21;12(7):e0181678. doi: 10.1371/journal.pone.0181678 (PMC5521830; doi:10.1371/journal.pone.0181678)

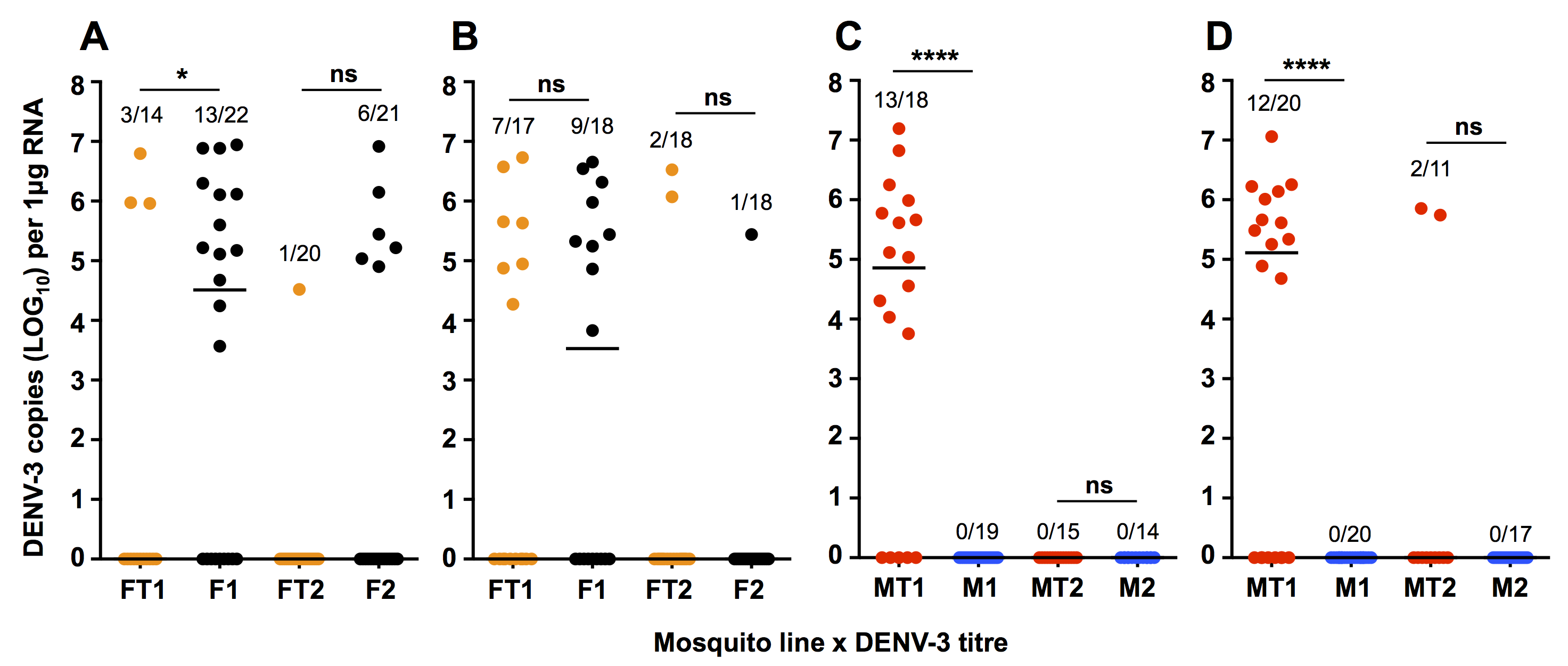

Supplement: S1 Fig — Prevalence of infection and DENV load for Ae. fluviatilis at 7 (A) and 14dpi (B), and Ae. aegypti at 7 (C) and 14dpi (D), as determined via RT-qPCR with absolute quantification. The figure shows data for two DENV-3 concentrations that were fed to mosquitoes: 1.9 x10^6^ pfu/mL (1), and 1.9 x 10^4^ pfu/mL (2). FT (orange)—Flu.Tet. F (black)—Flu. MT (red)—Mel.Tet. M (blue)—Mel. Prevalence data analysed by Fisher’s exact test. DENV load data analysed by Mann-Whitney U test. ns = P > 0.05, * = P < 0.05, **** = P < 0.0001. Black lines represent treatment medians. (TIFF) [file pone.0181678.s001.tiff]
